# Reversible Cerebral Vasoconstriction Syndrome in the Postpartum Period: A Systematic Review and Meta-Analysis

**DOI:** 10.3390/neurolint14020040

**Published:** 2022-05-31

**Authors:** Kimberly Pacheco, Juan Fernando Ortiz, Jashank Parwani, Claudio Cruz, Mario Yépez, Maja Buj, Mahika Khurana, Diego Ojeda, Alisson Iturburu, Alex S. Aguirre, Ray Yuen, Shae Datta

**Affiliations:** 1 School of Medicine, Colegio de Ciencias de la Salud, Universidad San Francisco de Quito, Quito 170901, Ecuador; cruzclaudio9014@gmail.com (C.C.); jose7diego@hotmail.com (D.O.); aguaxindeclaus@gmail.com (A.S.A.); 2 Department of Neurology, California Institute of Behavioral Neuroscience & Psychology, Fairfield, CA 94534, USA; 3 Neurology, Lokmanya Tilak Municipal Medical College, Mumbai 400022, India; parwanijashank@gmail.com; 4 School of Medicine, Colegio de Ciencias de la Salud, Universidad Católica Santiago de Guayaquil, Guayaquil 090615, Ecuador; marioayepez@gmail.com; 5 East Side Medical Practice, New York, NY 10075, USA; bujmaja@gmail.com; 6 Department of Public Health, University of California, Berkeley, CA 94720, USA; mahikakhurana@gmail.com; 7 School of Medicine, Colegio de Ciencias de la Salud, Universidad de Guayaquil, Guayaquil 090510, Ecuador; dra.alisson.iturburu@gmail.com; 8 Neurology Department, Larkin Community Hospital, Miami, FL 33143, USA; ryuen@auis.edu; 9 Neurology Department, NYU Langone Health, New York, NY 10017, USA; shae.datta@nyulangone.org

**Keywords:** RCVS, postpartum, hemorrhagic

## Abstract

(1) Background: Reversible cerebral vasoconstriction syndrome (RCVS) encompasses a clinical and radiological diagnosis characterized by recurrent thunderclap headache, with or without focal deficits due to multifocal arterial vasoconstriction and dilation. RCVS can be correlated to pregnancy and exposure to certain drugs. Currently, the data on prevalence of RCVS in the postpartum period is lacking. We aim to investigate the prevalence of RCVS in the postpartum period and the rate of hemorrhagic complications of RCVS among the same group of patients; (2) Methods: We conducted the metanalysis by using the Preferred Reporting Items for Systematic Reviews and Meta-Analyses (PRISMA), and Meta-Analyses and Systematic Reviews of Observational Studies in Epidemiology (MOOSE) protocol. To analyze the Bias, we used the Ottawa Newcastle scale tool. We included only full-text observational studies conducted on humans and written in English. We excluded Literature Reviews, Systematic Reviews, and Metanalysis. Additionally, we excluded articles that did not document the prevalence of RCVS in the postpartum period (3). Results: According to our analysis, the Prevalence of RCVS in the postpartum period was 129/1083 (11.9%). Of these, 51/100 (52.7%) patients had hemorrhagic RCVS vs. 49/101 (49.5%) with non-hemorrhagic RCVS. The rates of Intracerebral Hemorrhage (ICH) and Subarachnoid Hemorrhage (SAH) were (51.6% and 10.7%, respectively. ICH seems to be more common than.; (4) Conclusions: Among patients with RCVS, the prevalence in PP patients is relativity high. Pregnant women with RCVS have a higher recurrence of hemorrhagic vs. non-hemorrhagic RCVS. Regarding the type of Hemorrhagic RCVS, ICH is more common than SAH among patients in the postpartum period. Female Sex, history of migraine, and older age group (above 45) seem to be risk factors for H-RCVS. Furthermore, recurrence of RCVS is associated with a higher age group (above 45). Recurrence of RCVS is more commonly idiopathic than being triggered by vasoactive drugs in the postpartum period.

## 1. Introduction

Reversible cerebral vasoconstriction syndrome (RCVS) was first reported in 1962 by Call and Flemming [1]. It is a clinical and radiological diagnosis and is characterized by recurrent thunderclap headache, with or without focal deficits due to multifocal arterial vasoconstriction and dilation [2]. It’s incidence is unknown, but it has been seen in approximately 0.26% of patients presenting to an emergency headache clinic; hence it is considered a rare clinical entity [3]. It is more common in women around 42 years old (10–76 years) [4], and some studies show that 7–9% of the patients had RCVS in the postpartum period (within one month from delivery) [3].

Precipitating factors include cannabis, cocaine, LSD, binge alcohol consumption, nicotine patches, noradrenergic and selective serotonergic antidepressants, selective serotonin reuptake inhibitors, and nasal decongestants interferon-alpha, steroids, bromocriptine, triptans, cyclosporine, epinephrine, and ergots [2]. Other examples of precipitants are use of vasoactive drugs, headache disorders (exertional headache, migraine, primary thunderclap headache, benign sexual headache), intra or extracranial disorders (head trauma, spinal subdural hematoma, head and neck surgery, CSF hypotension), catecholamine-secreting tumors (glomus tumors, pheochromocytoma, bronchial carcinoid tumors), and vascular associations (fibromuscular dysplasia, endovascular procedures, cervical artery dissection, unruptured intracranial aneurysm, carotid endarterectomy) [5].

RCVS is diagnosed if a patient fulfils the following criteria: (a) segmental cerebral artery vasoconstriction seen on the Magnetic Resonance Angiography (MRA), (b) No evidence of subarachnoid hemorrhage, (c) Normal CSF analysis (protein, leukocytes, glucose), (d) Severe headache with/without neurological findings, (e) reversibility of angiographic abnormalities within 12 weeks [6].

The pathophysiology of RCVS can be attributed to the tone of the vascular vessels of the cerebral arteries, sympathetic overactivity, and breakdown of the blood-brain barrier [7]. The reversible nature of the disease is due to a transitory central vascular discharge, and both headache and vasoconstriction are explained by the cerebral blood vessel’s innervation (sensory afferents from the trigeminal nerve) [4]. The alteration of small vessels may result in a distal arteriolar reperfusion injury or rupture (hemispheric, subarachnoid, or lobar hemorrhage), and if the larger vessels are affected, it can lead to multifocal areas of watershed infarction and ischemia. There are other postulated mechanisms involving mitochondrial dysfunction with oxidative stress, genetic predisposition, and biochemical and hormonal factors [5].

To the best of our knowledge, there has not been a systematic review or a meta-analysis on RCVS on the postpartum period. Additionally, we want to calculate the prevalence RCVS among females in the postpartum period and investigate the pathophysiology of RCVS.

## 2. Materials and Methods

### 2.1. Protocol

This systematic review was conducted following the Preferred Reporting Items for Systematic Reviews and Meta-Analyses (PRISMA) and Meta-Analysis of Observational Studies in Epidemiology (MOOSE) reporting guidelines [8].

#### 2.1.1. Eligibility Criteria and Study Selection

We only included clinical trials conducted on humans and written in English. Animal studies were excluded. We excluded papers that did not fulfill the aims of our study. After screening the studies, we only included papers with one of the following characteristics: (1) Patients: Female patients in the Postpartum Period (2) Intervention: Prevalence of RCVS (3) Comparator: No controls; and (4) Outcomes: (a) Prevalence of RCVS (b) Frequency of ICH, Frequency of Intracerebral Hemorrhage (c) Frequency of SAH, Recurrence rate of RCVS. Two authors (J.F.O & M.Y) extracted the data independently. 

#### 2.1.2. Database and Search Strategy

We used the PubMed and Google Scholar databases for this systematic review. The search was conducted between 1 March 2022 and 15 March 2022. We used an advanced search strategy with the following terms: (RCVS [Title/Abstract]) AND (Postpartum [Title/Abstract]).

#### 2.1.3. Data Extraction and Analysis

We collected the following information from each paper: the methods, such as dose, duration, route of administration and number of participants, study design, and patient selection. We also extracted the main results, including the outcome measures and main limitations of each observational study.

#### 2.1.4. Bias Assessment

We used the Otawa-Newcastle Scale to assess the bias encountered in each study [9].

#### 2.1.5. Data Analysis

We used random effect model to make the prevalence analysis of this systematic review. For the data analysis, we used “Open Meta-Analysis Software (Englewood, NJ, USA)”, version 10.10. where we calculated the pool prevalence of RCVS in all women during the postpartum period with the 95% CI. We also determined the prevalence of the type of hemorrhage (SAH, ICH). Lastly, we calculated the recurrence rate of RCVS among females in the postpartum periodusing the Open Metanalysis Software [10].

## 3. Results

**Figure 1:**
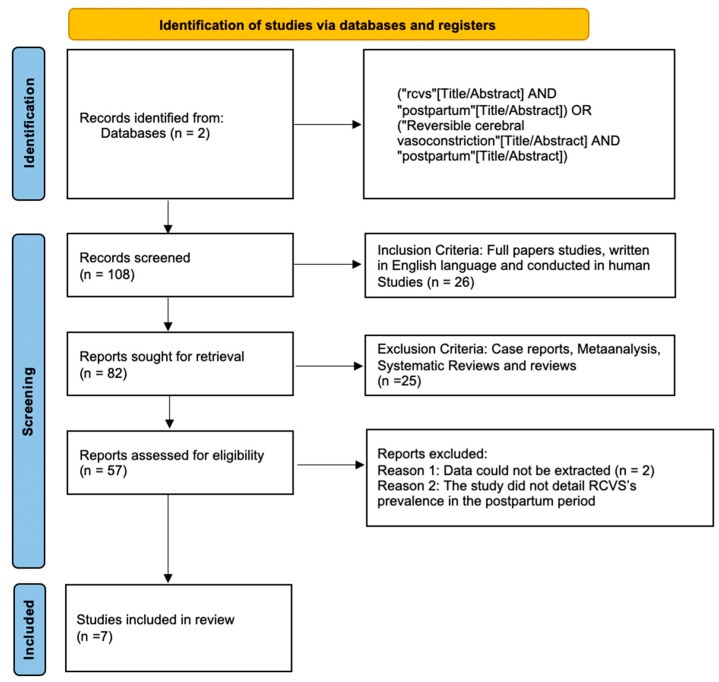
Flowchart of the study.

Figure 1 shows the results of the study using a PRISMA Flow chart.

Table 1 shows the frequency of ICH, SAH, and Recurrence of RCVS among patients in the postpartum period [11,12,13,14,15,16,17].

**Figure 2:**
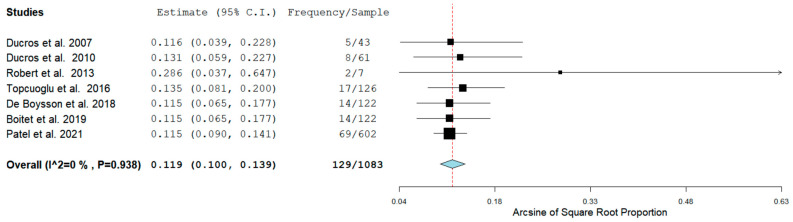
Pool Prevalence of RCVS in the postpartum period.

Seven studies detailed the prevalence of RCVS in the Postpartum period, while five studies reported the hemorrhagic rate of RCVS in the same group of patients. In four studies the rate of ICH RCVS and SAH RCVS was reported as well. Figure 2 shows the prevalence of RCVS in the Postpartum Period [11,12,13,14,15,16,17]. 

**Figure 3:**
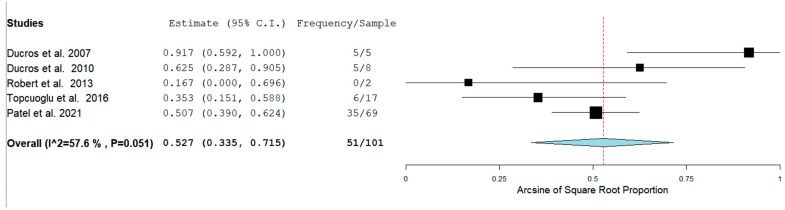
Rate of hemorrhagic RCVS in the Postpartum Period.

The Prevalence of RCVS in the Postpartum period in our analysis was 129/1083 (11.9%) across seven studies. RCVS could be either hemorrhagic or non-hemorrhagic. Figure 3 shows the rate of hemorrhagic RCVS in the Postpartum Period [11,12,13,14,17]. 

**Figure 4:**
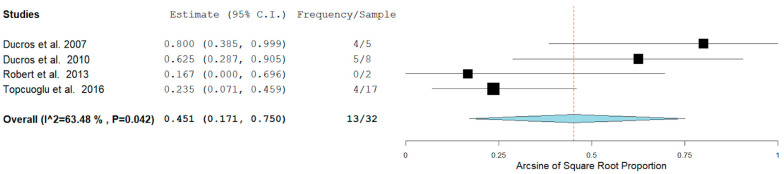
Rate of ICH among females with RCVS in the Postpartum Period.

**Figure 5:**
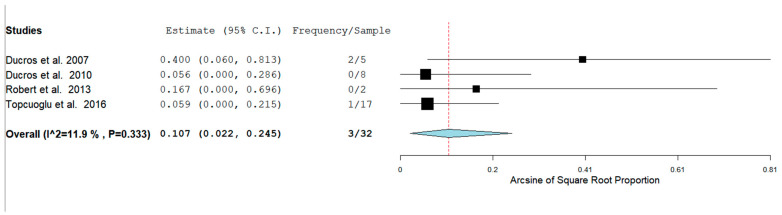
Rate of SAH among females with RCVS in the Postpartum Period.

In our analysis, 51/100 (52.7%) patients had hemorrhagic RCVS vs non-hemorrhagic RCVS 49/101 (49.5%). There are two types of hemorrhagic RCVS: Intraparenchymal or intracerebral (ICH) and subarachnoid hemorrhage (SAH). Figure 4 and Figure 5 show the rate of ICH and SAH among female patients with RCVS in the Postpartum period [11,12,13,17]. 

The rates of ICH and SAH were 51.6% and 10.7% respectively. ICH seems to be more common than SAH as a hemorrhagic complication. 

### Bias Analysis

The study by Patel et al. was limited because the retrospective design of this study causes selection bias. Additionally, it was done with hospitalized patients and did not consider outpatient settings, which could lead to misdiagnosis, delayed diagnosis, and the underestimation of true incidence. Finally, there were administrative reporting errors in the International Classification of Diseases-10 codes for RCVS [17].

Inconsistent imaging timing limited Tocuoglu et al.’s study model, and thus subacute findings may be biased toward symptomatic lesions. Besides, iatrogenic factors may influence lesion development and were not considered in the study [14]. Robert et al.’s study was limited by the retrospective design and the small cohort of patients [13]. Ducros et al.’s study was not free of bias because it was a prospective single-center observational study. They did not blind the observers of the neuroimaging data to the study’s hypotheses or the clinical status of the patient’s [12].

Boitet et al.’s study was limited because there were no regular prespecified intervals for the follow-up, and there was no standardized imaging paradigm for recurrent headaches. There is also an underestimation of the rate of R-RCVS because of a lack of systematic vascular imaging and blinding of the observers of neuroimaging data. Moreover, the study did not assess patients that avoided vasoconstrictors, so they could not calculate the relapse rate [15].

There were some limitations in De Boysson et al.’s study as well. Complete data retrieval is limited by a retrospective study design. The two cohorts (only French population) show differences from other published series, limiting the results’ extrapolation. Additionally, only reported patients were selected for the study, so it did not reflect real-life practice [16]. Finally, in the study by Ducros et al. the rate of RCVS was underdiagnosed because it was a single prospective center observational study with a small sample [11].

Table 2 shows the BIAS Analysis of this study by using the NewCastle-Ottawa Scale [11,12,13,14,15,16,17].

## 4. Discussion

### 4.1. Role of Sex and Pregnancy

RCVS is a syndrome with a clinical and radiographic diagnosis. Women seem to have a lower threshold for developing RCVS as compared to men. In the systematic review of Song et al. 81.2% of patients were women [18]. Compared to men, women diagnosed with RCVS are usually older, have more frequency of migraines, more use of serotonergic drugs, and clinical worsening; however, both men and women have similar discharge outcomes [14]. In the study of Song et al. the prevalence of women was higher than in our study (73.5%). Even so, unlike their research, our study only included observational studies, where PP RCVS was mentioned. 

Female reproductive hormones are considered to play an essential role in the pathogenesis of RCVS. Cerebral arteries express specific receptors for these hormones and are involved in altering the permeability of the blood-brain barrier [19]. It has been shown that estrogen reduces cerebral tone and inhibits central sympathomimetic activity via prostanoids, endothelial nitric oxide, and other molecular pathways [17]. While these factors may give rise to a higher incidence of RCVS, the female reproductive hormones do not play a significant role in the severity or course of the disease. Moreover, other non-hormonal elements can trigger PP RCVS, such as Soluble P1GF receptor (sFlt-1) and placental growth factor (PlGF). Finally, RCVS in men can be triggered by changes in the levels of prolactin, sympathomimetic amines, and oxytocin that occur during the sexual orgasm [19].

There are differences between PP and non-pregnant women. In Topcuoglu et al. (2016) study, women in the PP were younger and less likely to use vasoconstrictive drugs. There were no significant differences in the same study between pre and postmenopausal women or those with or without hysterectomy [19]. 

### 4.2. Hemorrhagic RCVS

RCVS can be hemorrhagic (Intracerebral and Subarachnoid hemorrhage) and non-hemorrhagic. Clinically, both groups have the same symptomatology but different frequencies of presentation. In most cases, patients initially present with only a thunderclap headache or a single acute headache; other presentations include focal deficit, seizures, elevated blood pressure, and cerebral infarction, which are more frequent in hemorrhagic RCVS [12]. Visual symptoms (blurred vision and photophobia) are more common in non-hemorrhagic RCVS and are often accompanied by vomiting or nausea [13].

Three studies have documented the rate of H RCVS among all types of patients with the syndrome. The rates of H-RCVS in Patel et al. Topcuoglu et al. and Ducros et al. were 43.3%, 43%, and 34%, respectively [12,14,17]. In our pooled analysis which only included females in the PP, the Hemorrhagic rate was 50% (51/101). Our results suggest that patients in the postpartum period could have a higher risk of presenting H-RCVS than all RCVS patients. 

Considering just the postpartum females, Ducros et al. found no significant higher incidence of H-RCVS (19% vs. 9%) [12]. However, Patel et al. and Topcuoglu et al. found a similar incidence of H-RCVS in the postpartum period [14,17]. In our pooled analysis, we found a rate of 9.58% of H-RCVS in the PP sample. 

The study by Ducros et al. Topcuoglu et al. and Patel et al. determined that female sex was an independent risk factor for ICH in patients with RCVS [12,14,17]. Additionally, they found migraine history to be another independent risk factor [12]. In contrast, Patel et al. inferred that patients between (45–64 years) and the older age group (>64 years) had a higher association of ICH compared to younger patients [17].

Regarding the type of bleeding in these studies, the rate of SAH was (5.88%) and (0%) in Topcuoglu et al. and Ducros et al. respectively [12,14]. In our pooled analysis, the rate of SAH among females in the PP was 9%. While the rate of ICH was (23.5%) and (62.5%) in Topcuoglu et al. and Ducros et al. studies, respectively [14], it was 40% among PP females in our pooled analysis.

### 4.3. Recurrence of RCVS

The exact rate of recurrence is unknown [20]. However, two studies have documented the rate of recurrent RCVS among all patients with the syndrome. The rates of R-RCVS in Chen et al. and Song et al. were 5.4% and 4.7%, respectively [18,20]. Chen et al. stated that the recurrence is associated with older age (53.8 ± 5.8 years) and a delay of 6 months to 7 years from the first to the second episode. Moreover, 8/9 cases were idiopathic, and 1/9 were triggered by vasoactive drugs [20]. In our study, we included only females in the postpartum period, and the recurrence rate among seven studies was 0%. Our results suggest that patients in the PP could have a lower risk of presenting R-RCVS as compared to all types of patients with the syndrome because most pregnancies occur at a younger age.

## 5. Conclusions

Among PP patients, the prevalence of RCVS is relativity high. Pregnant women with RCVS have a higher recurrence of hemorrhagic vs. non-hemorrhagic RCVS. With regard to the type of Hemorrhagic RCVS, ICH is more common than SAH among patients in the Postpartum Period.

Female sex, migraine history, and older group (above 45) of patients seem to be risk factors for H-RCVS. At the same time, recurrence of RCVS may be associated with a higher age group (above 45). Recurrence of RCVS is more commonly idiopathic than being triggered by vasoactive drugs in the Postpartum Period.

## Figures and Tables

**Table 1:** The frequency of ICH, SAH, and Recurrence of RCVS among patients in the postpartum period.

Author and Year of Publication, Country	Study Design	No. of Patients	Mean Age	Associated Conditions	Prevalence of RCVS in the PP among Females	Hemorrhage among RCVS Patients in the PP	Recurrence among RCVS Patients in the PP
Ducrose et al. (2007) France [11]	Prospective single center observational study	N = 67 (F: 43 and M: 24)	42.5 ± 11.8 (F: 46.9 ± 11.5)	**Postpartum period (8%)**, vasoactive substance (55%), none (37%)	5/43	5/5 (ICH 4/5 and SAH 2/5)	Not reported
Ducrose et al. (2010) France [12]	Prospective cohort	N = 89 (F: 61 and M: 28)	RCVS With ICH: 46.6 ± 11.0 RCVS Without ICH: 41.6 ± 11.6	**Postpartum period (8.98%)**, vasoactive substance (51.68), migraine (26.96%), HTA (11.23%)	8/61	5/8 (ICH 5/8 and SAH 0/8)	Not reported
Robert et al. (2013) Switzerland [13]	Retrospective multi-center review	N = 10 (F: 7 and M: 3)	46	**Postpartum period (29%)**, Migraine (28%), HTA (71%), vasoactive substances (57%)	2/7	0/2 (ICH 0/2 and SAH 0/2)	Not reported
Topcuoglu et al. (2016) United States [14]	Single center restrospective study	N = 162 (F:126 and M: 36)	44 ± 13	**Postpartum period (Hem: 9% no Hem: 11%)**, vasoconstrictive drugs (Hem: 61% no Hem: 59%), Physiological/Idiopathic (Hem: 31% no Hem: 29%), HTA (Hem: 42% no Hem: 3	17/126	6/17 (ICH 4/17 and SAH 1/17)	Not reported
De Boysson et al. (2018) France [15]	Comparative study	N = 173 (F: 122 and M: 51)	44	**Postpartum period (8.09%)**, Migraine (32.37%), tobacco use (35.83%), HTA (15.02%), vasoactive substances (49.13%)	14/122	Not reported	Not reported
Boitet et al. (2019) France [16]	Prospective single center observational study	N = 173 (F: 122 and M: 51)	Not reported	**Postpartum period (8.18%)**, vasoactive substance (48.53%), none (43.29%)	14/122	Not reported	0/14
Patel et al. (2021) United States [17]	Retrospective observational study (data base)	N = 799 (F: 602 and M: 197)	46.3 ± 0.8	**Postpartum period (11.5%)**, Migraine (22%), HTA (51.8%), Inflammatory disorders (18.2%), pregnancy (6.5%), pregnancy HTA (6.6%)	69/602	35/69	Not reported
Totales		1083/1473 (73.5%)					

**Table 2:** BIAS Analysis of this study.

Study	Newcastle-Ottawa Scale			Overall Risk of Bias
	Selection (max 4)	Comparability (max 2)	Outcome/Exposure (max 3)	
Ducrose et al. France, 2007	***	**	**	**Moderate**
Ducrose et al. France, 2010	***	**	**	**Moderate**
Robert et al. Switzerland, 2013	*	*	*	**High**
Topcuoglu et al. United States, 2016	***	**	**	**Moderate**
De Boysson et al. France, 2018	***	**	**	**Moderate**
Boitet et al. France, 2019	***	**	**	**Moderate**
Patel et al. United States, 2021	***	**	**	**Moderate**
